# Synthesis and characterization of benzodithiophene and benzotriazole-based polymers for photovoltaic applications

**DOI:** 10.3762/bjoc.12.160

**Published:** 2016-08-01

**Authors:** Desta Gedefaw, Marta Tessarolo, Margherita Bolognesi, Mario Prosa, Renee Kroon, Wenliu Zhuang, Patrik Henriksson, Kim Bini, Ergang Wang, Michele Muccini, Mirko Seri, Mats R Andersson

**Affiliations:** 1 Future Industries Institute, University of South Australia, Mawson Lakes, South Australia 5095, Australia; 2 Department of Chemistry and Chemical Engineering, Polymer Technology, Chalmers University of Technology, Göteborg SE-412 96, Sweden; 3 National Research Council (CNR) – Institute of Nanostructured Materials (ISMN), Via P. Gobetti, 101, 40129 Bologna, Italy; 4 Laboratory MIST E-R, Via P. Gobetti, 101, 40129 Bologna, Italy; 5 National Research Council (CNR) − Institute of Organic Synthesis and Photoreactivity (ISOF), Via P. Gobetti, 101, 40129 Bologna, Italy

**Keywords:** alkyl side chains, benzodithiophene, bulk heterojunction solar cells, 2D conjugated polymers, fluorinated benzotriazole

## Abstract

Two high bandgap benzodithiophene–benzotriazole-based polymers were synthesized via palladium-catalysed Stille coupling reaction. In order to compare the effect of the side chains on the opto-electronic and photovoltaic properties of the resulting polymers, the benzodithiophene monomers were substituted with either octylthienyl (PTzBDT-1) or dihexylthienyl (PTzBDT-2) as side groups, while the benzotriazole unit was maintained unaltered. The optical characterization, both in solution and thin-film, indicated that PTzBDT-1 has a red-shifted optical absorption compared to PTzBDT-2, likely due to a more planar conformation of the polymer backbone promoted by the lower content of alkyl side chains. The different aggregation in the solid state also affects the energetic properties of the polymers, resulting in a lower highest occupied molecular orbital (HOMO) for PTzBDT-1 with respect to PTzBDT-2. However, an unexpected behaviour is observed when the two polymers are used as a donor material, in combination with PC_61_BM as acceptor, in bulk heterojunction solar cells. Even though PTzBDT-1 showed favourable optical and electrochemical properties, the devices based on this polymer present a power conversion efficiency of 3.3%, considerably lower than the efficiency of 4.7% obtained for the analogous solar cells based on PTzBDT-2. The lower performance is presumably attributed to the limited solubility of the PTzBDT-1 in organic solvents resulting in enhanced aggregation and poor intermixing with the acceptor material in the active layer.

## Introduction

Over the past decades the research on bulk heterojunction (BHJ) polymer solar cells (PSCs) has been intensified due to the attractive perspectives of producing lightweight and flexible devices via a scalable printing technology at low-cost. The active layer consists of a blend of π-conjugated polymer (electron donor) and fullerene derivative (electron acceptor) sandwiched between two electrodes (anode and cathode) [1–4]. Noticeable achievements have been recorded in terms of the power conversion efficiency (PCE) of lab-scale single junction BHJ PSCs surpassing the 10% milestone. It has also been possible to achieve improved PCE by using multi-junction structures (e.g., tandem) [5–8]. The continued development of new active materials with desired properties, understanding of nanoscale morphology and device architecture is expected to push the PCE to even higher value, offering promising perspectives for this technology [9–12].

Despite the different aspects, the properties of the donor polymers remain one of the most important factors on the overall performance of a BHJ device. Specifically, an ideal donor polymer is usually designed to have sufficient solubility in common organic solvents, good stability in air, a suitable bandgap for an effective light harvesting, proper charge transport properties, suitable HOMO and LUMO energy levels compatible with the acceptor material [13] and an excellent film-forming capability. In search of materials endowed with these properties, a huge number of new polymer structures have been designed, synthesized and used in fabricating efficient BHJ solar cells.

Among these donor polymers, copolymers based on widely known structural units such as benzo[1,2-*b*:4,5-*b*']dithiophene (BDT) and 5,6-difluoro-2*H*-benzo[*d*][1,2,3]triazole (Tz) have attracted much attention and effectively employed in BHJ PSCs due to their intrinsic advantages, potentials and versatility [14–15]. Thanks to the desirable properties such as structural rigidity, planarity, extended π-conjugation length and favorable interchain π–π stacking, BDT is a widely used electron-rich monomer. Moreover, alkyl or aryl groups can easily be introduced to BDT basic units as side groups to finely tune the properties of the resulting polymers, not only in terms of solubility but also contributing, for example, to extend the π-conjugation from the backbone to the lateral substituent (2D π-conjugated systems), thus leading to a bandgap reduction and higher charge carrier mobilities [15–18]. On the other hand, the Tz moiety, usually sandwiched between adjacent thiophene spacers to limit the inter-monomers steric hindrance, is a moderately weak electron-deficient unit that can be easily synthesized and its properties can be finely modulated by attaching groups on the reactive nitrogen atom of the triazole ring [15,19–20].

**Scheme 1:**
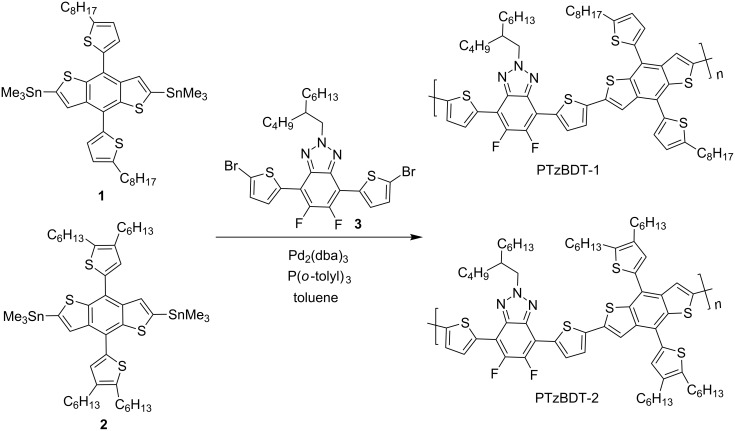
Stille cross coupling reaction for the synthesis of PTzBDT-1 and PTzBDT-2.

Here we report the synthesis and characterization of two novel donor polymers, PTzBDT-1 and PTzBDT-2 (Scheme 1), based on Tz and BDT moieties. The Tz ring was substituted with an asymmetrically branched alkyl side chain and sandwiched between two thiophene rings. The chemical structure of the Tz based monomer was made to be the same in both polymers for the comparative study. On the other hand, the BDT monomers used for the synthesis of PTzBDT-1 and PTzBDT-2 were substituted with either 2-octylthienyl (BDT-1) or 2,3-dihexylthienyl (BDT-2) as side groups, respectively. As a consequence of this fine structural modification on the BDT moiety, useful information on the effect of the different alkylthiophene side chains on the properties of the resulting pristine and blended films are collected and discussed.

Solution-processed BHJ PSCs using PTzBDT-1 or PTzBDT-2 as electron-donor materials and PC_61_BM as electron-acceptor counterpart were fabricated, optimized, and fully characterized. PCEs of 3.3% and 4.7% were achieved for PTzBDT-1 and PTzBDT-2 based devices, respectively, likely suggesting a different BHJ self-organization as a consequence of the different material properties induced by the alkyl substitution on the aromatic side groups.

## Results and Discussion

### Synthesis of the polymers

Scheme 1 shows the synthesis of the two polymers. The BDT (**1** and **2**) and Tz (**3**) based monomers were synthesized following literature procedures [21–22]. Thus, Stille cross-coupling reaction between the BDT and Tz based monomers gave the desired polymers in excellent yield. The molecular weights of the polymers were determined using size exclusion chromatography and the results are summarized in Table 1. PTzBDT-2 showed a higher molecular weight (*M*_n_ = 41.7 kDa) due to the two *n*-hexyl solubilizing alkyl side chains per thiophene attached to the BDT core unit. On the contrary, PTzBDT-1 with a relatively lower content of alkyl side chain (an *n*-octyl side chain per thiophene attached on the BDT) showed a relatively limited solubility resulting in a slightly lower molecular weight (*M*_n_ = 20.2 kDa). In fact, due to the limited solubility of PTzBDT-1, chlorobenzene was used as an extraction solvent to collect it from the extraction thimble at the polymer purification stage while PTzBDT-2, was extracted with chloroform thanks to its better solubility.

**Table 1:** Summary of the optical and electrochemical properties of PTzBDT-1 and PTzBDT-2.

Polymer	*M*_n_^a^ [kDa]	PDI	Solution			Thin-film			*E*_HOMO_ [eV]	*E*_LUMO_ [eV]

			λ_max_ [nm]	λ_onset_ [nm]	*E*_gap_^opt b^ [eV]	λ_max_ [nm]	λ_onset_ [nm]	*E*_gap_^opt b^ [eV]		

PTzBDT-1	20.2	4.40	550, 598	633	1.96	553, 598	646	1.92	−5.94	−3.25
PTzBDT-2	41.7	2.53	530, 574	605	2.05	536, 580	636	1.95	−5.86	−3.21

^a^Determined by GPC relative to polystyrene standards using 1,2,4-trichlorobenzene as eluent. ^b^*E*_gap_^opt^ = 1240/λ_onset_.

### Optical and electrochemical properties

**Figure 1:**
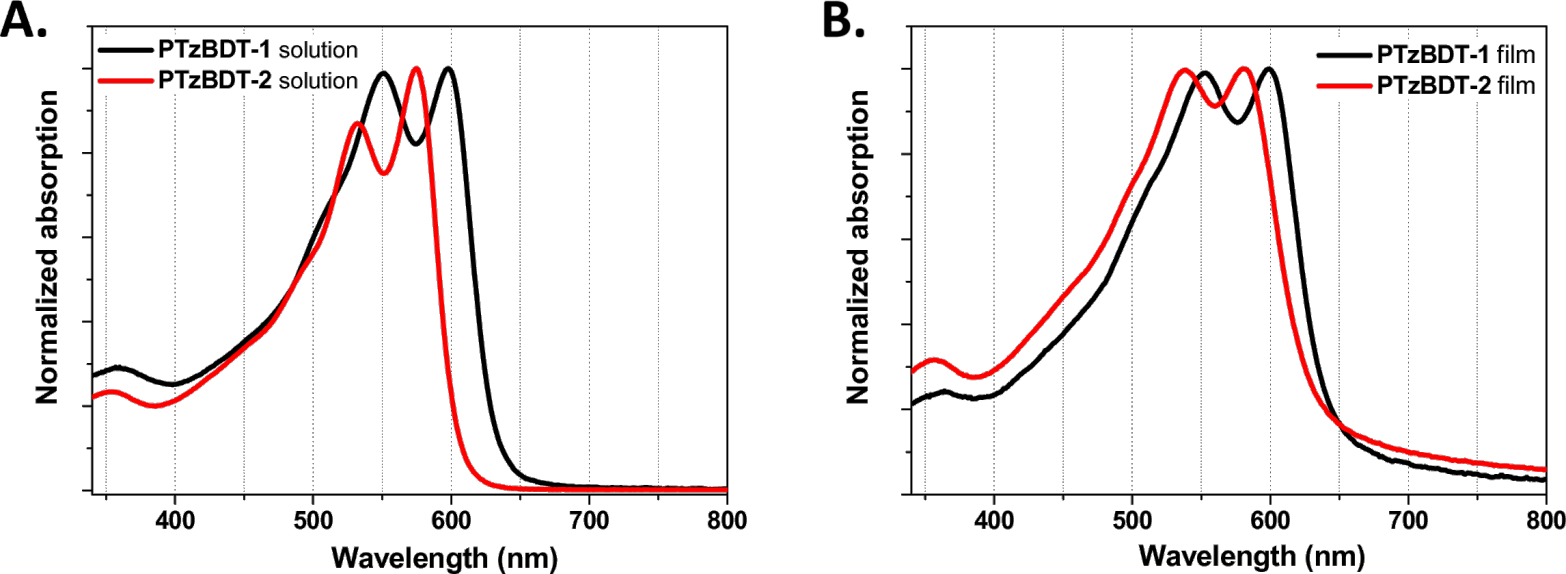
UV–visible absorption spectra of the pristine PTzBDT-1 and PTzBDT-2 (A) in chlorobenzene and chloroform, respectively and thin film processed from chlorobenzene (PTzBDT-1) and chloroform (PTzBDT-2) (B).

The UV–visible absorption spectra of the pristine PTzBDT-1 and PTzBDT-2 polymers in dilute solution (in chlorobenzene and in chloroform, respectively) and thin films are reported in Figure 1. The detailed absorption data are summarized in Table 1.

Both polymers show a modest peak at 360 nm, likely due to the thiophene side groups linked to the BDT unit [17]. The two evident bands between 500 and 650 nm are likely ascribed to intramolecular charge-transfer-like interactions between the monomers and interchain interactions [23–24], respectively. However, despite the similar molecular structures, the different absorption spectra of PTzBDT-1 and PTzBDT-2 confirm the crucial role of the aryl side groups on the aggregation, in solution and solid state, of the polymer chains.

The solution absorption spectra of the polymers (Figure 1A) exhibit an evident red-shift (~20 nm) of the λ_max_ of PTzBDT-1 compared to PTzBDT-2 (550/598 nm and 530/574 nm, respectively). Moreover, PTzBDT-1 shows a broader spectrum in comparison to PTzBDT-2 (Figure 1A), as also confirmed by the corresponding absorption onset values (λ_onset_) of 633 nm and 605 nm, respectively. Analogously to our previous work [21], it is reasonable to assume that for PTzBDT-2 (Scheme 1), the two *n*-hexyl side chains placed on the thiophene side groups could sterically interact with the Tz unit, probably leading to a partial twisting of the polymer backbone responsible for the observed trend.

This hypothesis is further confirmed if we consider the absorption onset values (λ_onset_) of the corresponding films (Table 1 and Figure 1B). Interestingly, the red shift magnitude (Δλ_onset_ = λ_onset_^film^ − λ_onset_^solution^) for PTzBDT-1 and PTzBDT-2 is 13 nm and 31 nm, respectively, indicating different intra- and intermolecular interactions likely induced by the alkyl side chain substitution [25–26]. As expected, the twisting of PTzBDT-2 is favored in dilute solution, while in film, where stronger intermolecular interactions take place, the polymer chains are likely forced to adopt a more planar conformation, in perfect agreement with the significantly red-shifted onset (Δλ_onset_ = 31 nm).

Diversely, PTzBDT-1 (Scheme 1) has a single *n*-octyl side chain linked to the thiophene ring, which should promote the solubility of the polymer without interacting with the adjacent Tz unit. As a result, a more planar conformation is expected for PTzBDT-1, thus allowing a partial pre-aggregation of the polymer chains in solution as confirmed by the relatively small Δλ_onset_ for PTzBDT-1 (13 nm). These findings combined with the still different film absorption spectra, suggest a different self-organization of the polymer chains, as supported by additional optical, electrical and morphological investigations of PTzBDT-1 and PTzBDT-2 based blends (vide infra).

**Figure 2:**
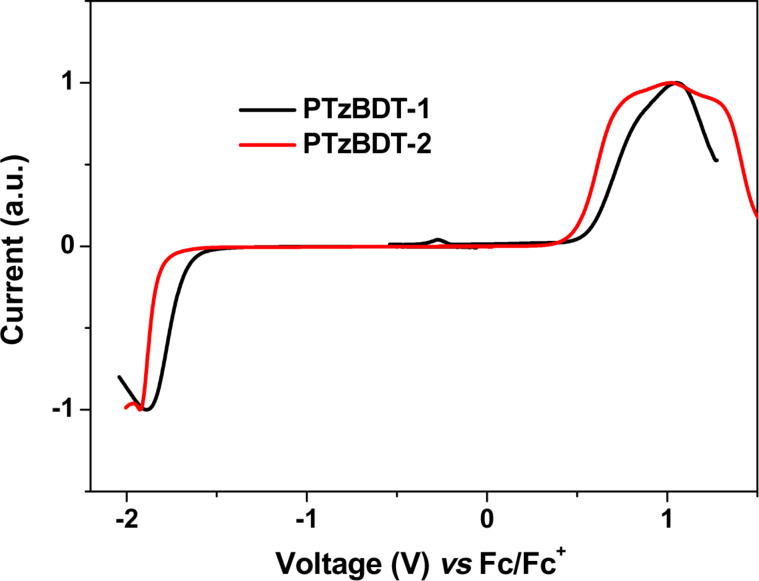
Square wave voltamogramme of PTzBDT-1 and PTzBDT-2.

The HOMO and LUMO energy levels of the polymer films were estimated by square wave voltammetry (SWV, Figure 2) using the oxidation and reduction peak values, respectively. As shown by the square wave voltammograms, the electrochemical oxidation shoulder peaks of PTzBDT-1 and PTzBDT-2 are located at 0.81 V and 0.73 V, respectively. As a result the estimated HOMO energy levels, calculated using the relation *E*_HOMO_ = −(*E*_ox_ + 5.13) [27], are −5.94 eV and −5.86 eV for PTzBDT-1 and PTzBDT-2, respectively. Similarly, the reduction peak potentials of PTzBDT-1 and PTzBDT-2 are located at −1.88 V and −1.92 V, respectively, resulting in LUMO energies of −3.25 and −3.21 eV, (*E*_LUMO_= −(*E*_red_ + 5.13)) [27]. Note that the number of alkyl chains linked to the aromatic side groups of the polymers are not expected to significantly affect the π-electron density distribution and thus the energetic properties of the two polymers [21], however the subsequent different organization in the solid state might be the main factor responsible for the observed variation of the HOMO and LUMO energy levels, which is in perfect agreement with the different optical properties. Interestingly, the deep HOMO energies of both polymers would result in devices with a high open circuit voltage (V_OC_), according to the difference LUMO_ACCEPTOR_ − HOMO_DONOR_ [28]. Good air stability is also expected from these polymers as their HOMO energies are in an ideal range [29]. On the other hand, the slightly raised LUMO observed in both polymers is expected due to the moderately weak electron withdrawing nature of benzotriazole.

It should be noted that there is a discrepancy between the bandgaps derived from electrochemical and optical measurements of PTzBDT-1 and PTzBDT-2. This incongruence can be ascribed to the different method employed for the measurements. Indeed, in the first case ionized states are generated, while after light absorption the excited state is based on electrons and holes electrostatically bound [30]. Moreover, an energetic barrier between the electrode surface and the polymer film can further contribute to increase the electrochemically derived energies [31]. Finally, the resulting thin-film quality, and subsequent polymer self-organization, prepared over the electrode or over a flat substrate might be different, reflecting possible variations.

### Photovoltaic properties

**Figure 3:**
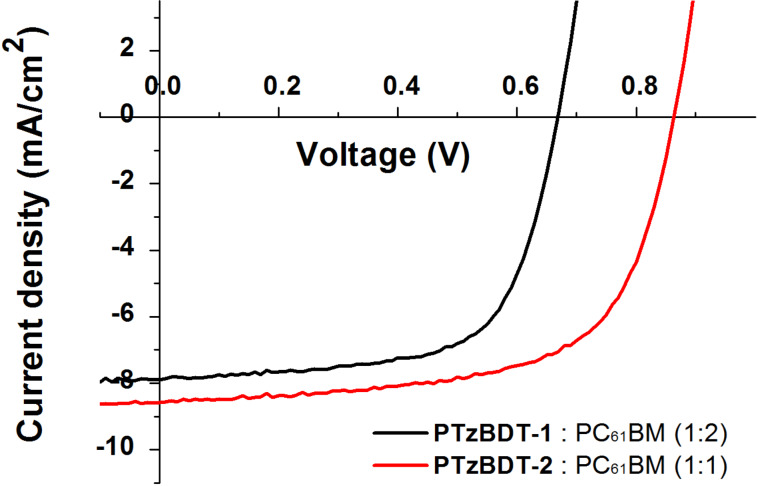
*J*–*V* plots, measured under standard illumination (AM1.5G, 100 mW/cm^2^), of PTzBDT-1: PC_61_BM and PTzBDT-2: PC_61_BM BHJ based devices.

A set of BHJ solar cells, using PTzBDT-1 and PTzBDT-2 as donors and PC_61_BM as an acceptor counterpart, were fabricated and characterized in order to evaluate the impact of the alkyl substitution on the resulting photovoltaic performance. Devices with standard configuration, glass/ITO/PEDOT:PSS/active layer/LiF/Al, were used. The PTzBDT-1 and PTzBDT-2 based active layers were spin-coated respectively from 1,2,4-trichlorobenzene (TCB) and 1,2-dichlorobenzene (ODCB) solutions (best solvents in terms of solubility and thin-film quality for each polymer) without the need of additional processing solvent additives. All the details for the fabrication and characterization of the devices are reported in the experimental section. The photovoltaic responses including *V*_OC_, short circuit current density (*J*_SC_), fill factor (FF), and PCE of optimized devices are summarized in Table 2. The corresponding current density–voltage (*J*−*V*) plots of the most efficient devices, measured under standard illumination (AM1.5G, 100 mW/cm^2^), are shown in Figure 3.

**Table 2:** PV characteristics of optimized PTzBDT-1:PC_61_BM and PTzBDT-2:PC_61_BM BHJ devices. The reported results are averaged over 4 solar cells.

Donor:acceptor ratio [wt/wt]	Solvent	Thickness [nm]	Annealing [°C]	*J*_SC_ [mA/cm^2^]	*V*_OC_ [V]	FF [%]	PCE [%]

PTzBDT-1:PC_61_BM (1:2)	TCB	100	110^a^	7.6	0.67	64	3.3
PTzBDT-2:PC_61_BM (1:1)	ODCB	90	–	8.6	0.86	64	4.7

^a^Annealing time: 10 min.

The optimal polymer:fullerene ratio was 1:2 and 1:1 (wt/wt) for PTzBDT-1 and PTzBDT-2 based blends, respectively. By varying the amount of the donor content in the BHJ blends, a reduction of the PCEs was observed (Table 3).

**Table 3:** PV characteristics of optimized PTzBDT-1:PC_61_BM and PTzBDT-2:PC_61_BM BHJ devices using different donor:acceptor ratios and processing conditions.

Active blend	D:A ratio [wt/wt]	Solvent^a^	Annealing [°C]	*J*_SC_ [mA/cm^2^]	*V*_OC_ [V]	FF [%]	PCE [%]

PTzBDT-1:PC_61_BM	1:1	TCB	No ann.	8.0	0.63	42	2.1
	1:2	TCB	110^b^	7.4	0.66	62	3.0

PTzBDT-2:PC_61_BM	1:1	ODCB	110^b^	8.3	0.84	60	4.2
	1:2	ODCB	No ann.	6.6	0.87	65	3.7
	1:2	ODCB	110^b^	4.7	0.85	60	2.4

^a^Additional solvents have been also tested for each polymer, however the resulting films were unhomogeneous with a poor morphology. For this reason BHJ Devices were not fabricated; ^b^annealing time: 10 min.

Additional experiments to further enhance the device performance, for example by testing different processing solvents, thicknesses, annealing temperatures and annealing times (relevant examples are reported in Table 3), were unsuccessfully carried out.

**Figure 4:**
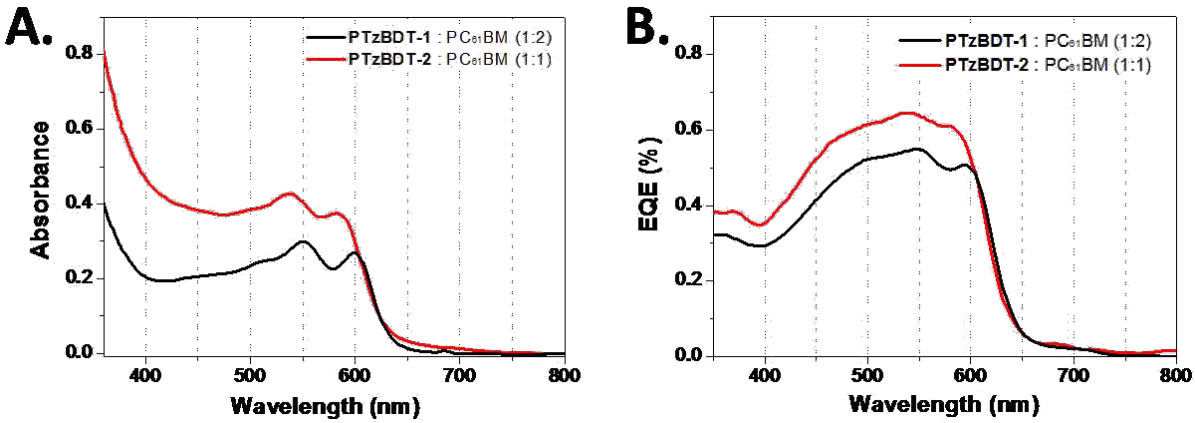
A) UV–vis absorption spectra and, B) EQE plots of optimized PTzBDT-1/PTzBDT-2:PC_61_BM based devices.

The optimized BHJ solar cells exhibit PCEs of 3.3% and 4.7%, respectively for 1:2 (wt/wt) PTzBDT-1:PC_61_BM and 1:1 (wt/wt) PTzBDT-2:PC_61_BM films. The PTzBDT-1 based device shows relatively low performance with a *V*_OC_ = 0.67 V, *J*_SC_ = 7.6 mA/cm^2^ and FF = 64%, while the device based on PTzBDT-2 exhibits a *V*_OC_ = 0.86 V, *J*_SC_ = 8.6 mA/cm^2^ and FF = 64%. The *V*_OC_ and *J*_SC_ values, which simultaneously increase from PTzBDT-1 to PTzBDT-2, are the main parameters responsible for the different photovoltaic responses. Interestingly, the FF is identical for both films (64%), indicating suitable charge transport properties within the blends. By comparing the *V*_OC_ values an increase of 0.19 V is observed passing from PTzBDT-1 to PTzBDT-2. This difference, despite the deeper electrochemically derived HOMO energy levels of PTzBDT-1 (Table 1), could be ascribed to the impact of the alkyl substitution of the thiophene ring (side group) on the chemico-physical (e.g., solubility) and film-forming properties of the corresponding polymer based blend, likely influencing the donor:acceptor phase segregation, molecular aggregation/distances and interfacial energetics, all factors strongly related to the resulting *V*_OC_ [21]. The improved *J*_SC_ (~15%) of the PTzBDT-2:PC_61_BM device in comparison to that based on PTzBDT-1 might be ascribed to the different optical property of the blends (Figure 4A). In particular, despite a comparable shape, the intensity of the absorption profiles, related to the donor content in the blend and responsible for the light harvesting and exciton generation, are significantly different reflecting the trend of the generated photocurrents. Interestingly, the absorption spectra of the optimized active blends present similar features observed for pristine materials. Indeed, despite the presence of PC_61_BM, the relative maxima of PTzBDT-2 are slightly blue-shifted in comparison to PTzBDT-1 likely reflecting the different conformation and or twisting of the polymer backbone as previously discussed.

These results suggest that, beside structural factors, other fundamental aspects such as the slightly better solubility of PTzBDT-2 (double alkyl substitution on the thiophene ring) in comparison to PTzBDT-1, not only allows a higher donor content in the blend (enhanced light absorption) but also seems to be crucial in terms of precipitation/segregation kinetics during the deposition/drying process of the active blend, strongly influencing the self-organization, the quality and thus the morphological features of the resulting BHJ film (see below).

The external quantum efficiency (EQE) spectra of optimized PTzBDT-1 and PTzBDT-2 based devices (the same thickness as the best devices), shown in Figure 4B, are consistent with the absorption spectra of the corresponding blends (Figure 4A). Specifically, the EQE responses of PTzBDT-1:PC_61_BM and PTzBDT-2:PC_61_BM based devices reach the maxima of 55% (at 546 nm) and 64% (at 536 nm), respectively, in perfect agreement with the first relative absorption maxima of the corresponding films. The integrated currents from the EQE plots are in good agreement, within a ~10% experimental error, with the experimental values obtained from *J*–*V* measurements.

**Figure 5:**
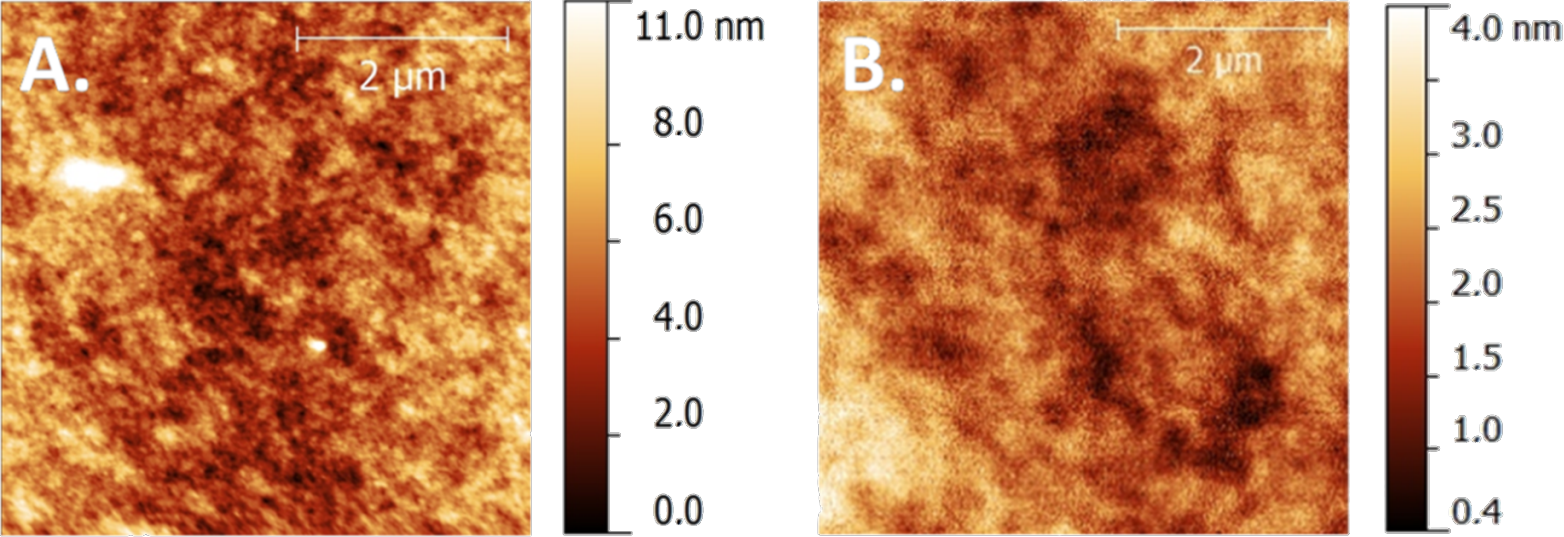
AFM images (size: 5 µm × 5 µm) of: A) 1:2 (wt/wt) PTzBDT-1:PC_61_BM (RMS of ~1.5 nm) and, B) 1:1 (wt/wt) PTzBDT-2:PC_61_BM (RMS of ~0.5 nm) blends.

In order to further investigate the impact of the side chain architecture of PTzBDT-1 and PTzBDT-2 on the solar cell output parameters, we compare the morphological differences of the corresponding optimized blends by tapping-mode atomic force microscope (AFM) (Figure 5).

The surface morphology of the films is quite different, reflecting the trend of the photovoltaic responses. In particular, the topographic image of the 1:2 (wt/wt) PTzBDT-1:PC_61_BM film (Figure 5A) is characterized by an almost featureless surface with randomly oriented and poorly defined domains suggesting a suboptimal phase segregation of the donor:acceptor blend. Diversely, the AFM image of 1:1 (wt/wt) PTzBDT-2:PC_61_BM blend (Figure 5B) seems to be based on more structured and defined domains indicating a higher donor:acceptor intermixing combined with finely ordered and aggregated polymeric domains, in perfect agreement with the improved photovoltaic performance. This better self-organization of the PTzBDT-2:PC_61_BM blend seems in contrast with the intrinsic structural features of the polymer, where the double alkyl substitution is likely responsible for the partial twisting of the polymer backbone, however its enhanced solubility should promote the quality of the resulting thin films, highlighting the key role of the side chains to reach the best compromise between solubility and molecular packing in the solid state for this class of polymers.

## Conclusion

In conclusion, we reported the synthesis and characterization of two high bandgap polymers based on BDT and Tz units. We showed that the aryl substitution pattern on BDT is an important factor for the reorganization of the polymer in the solid state, affecting the optical and electrochemical properties of the pristine polymer thin films as well as the photovoltaic performance of the corresponding solar cells. Indeed, the polymer with dihexylthiophene substituted BDT (PTzBDT-2) showed better solubility and hence formed a well-developed nanomorphology when mixed with PC_61_BM in comparison to the analogous octylthiophene substituted polymer (PTzBDT-1). As the result, PTzBDT-2 gave a PCE of 4.7% when combined with PC_61_BM. On the other hand, the polymer with octylthiophene substituted BDT (PTzBDT-1) showed a PCE of 3.3% likely due to the slightly lower solubility responsible for the generation of a suboptimal BHJ morphology.

## Experimental

### General

Size exclusion chromatography was performed on Waters Alliance GPCV2000 with a refractive index detector, with columns: Waters Styragel^®^ HT 6E×1, Waters Styragel^®^ HMW 6E×2. The eluent was 1,2,4-trichlorobenzene and the measurment was performed at 135 °C. The concentration of the samples was 0.5 mg/mL, which was ﬁltered (ﬁlter: 0.45 μm) prior to the analysis. The relative molecular masses were calculated by calibration relative to polystyrene standards.

Square-wave voltammetric measurements were carried out on a CH-Instruments 650A Electrochemical Workstation. As described in [27], a three-electrode setup consisting of platinum wires, both as working electrode and counter electrode, and a Ag/Ag^+^ quasi reference electrode were used. A 0.1 M solution of tetrabutylammonium hexafluorophosphate (Bu_4_NPF_6_) in anhydrous acetonitrile was used as supporting electrolyte. The polymers were deposited onto the working electrodes from chloroform solutions. The electrolyte was bubbled with nitrogen gas prior to each experiment. During the scans, nitrogen gas was flushed over the electrolyte surface. After each experiment, the system was calibrated by measuring the ferrocene/ferrocenium (Fc/Fc^+^) redox peak. The HOMO and LUMO energy levels of the polymers and electron acceptors were calculated from the peak values of the third scans by setting the oxidative peak potential of Fc/Fc^+^ vs the normal-hydrogen electrode (NHE) to 0.630 V and the NHE *vs* the vacuum level to 4.5 V [27].

### Synthesis of the polymers

As described in [17], the polymers were synthesized according to the following synthetic procedures.

#### Synthesis of PTzBDT-1

(4,8-Bis(5-octylthiophen-2-yl)benzo[1,2-*b*:4,5-*b*']dithiophene-2,6-diyl)bis(trimethylstannane) (**1**, 0.208 g, 0.23 mmol) and 4,7-bis(5-bromothiophen-2-yl)-2-(2-butyloctyl)-5,6-difluoro-2*H*-benzo[*d*][1,2,3]triazole (**3**, 0.15 g, 0.23 mmol) were dissolved in toluene (10 mL) and degassed with N_2_ gas for 10 minutes. Pd_2_(dba)_3_ (4.2 mg, 2 mol %) and P(*o*-tolyl)_3_ (6.3 mg, 9 mol %) were added and purged with nitrogen gas for 25 minutes. The reaction mixture was heated at 90 °C for 40 min. The polymer solution was then added to methanol and the solid formed was collected by filtration. The polymer was re-dissolved in chlorobenzene by heating at 60 °C for 1 hour and 10% aqueous solution of sodium diethyldithiocarbamate trihydrate (100 mL) was added and stirred at room temperature overnight. The chlorobenzene soluble portion was separated and washed with distilled water three times. The chlorobenzene solution was reduced to small volume and then added to methanol. The solid was collected and then purified by soxhlet extraction using methanol, hexane, diethyl ether, dichloromethane and chloroform. Finally, the polymer that goes into chlorobenzene was collected and the volume was reduced and precipitated by adding on methanol. The polymer was collected by filtration, dried in a vacuum oven at 40 °C overnight to give a black solid (149 mg).

#### Synthesis of PTzBDT-2

(4,8-Bis(4,5-dihexylthiophen-2-yl)benzo[1,2-*b*:4,5-*b*']dithiophene-2,6-diyl)bis(trimethylstannane) (**2**, 0.095 g, 0.147 mmol) and 4,7-bis(5-bromothiophen-2-yl)-2-(4-((2-butyloctyl)oxy)butyl)-5,6-difluoro-2*H*-benzo[*d*][1,2,3]triazole (**3**, 0.15 g, 0.147 mmol) were dissolved in toluene (8 mL) and degassed with N_2_ gas for 10 minutes. Pd_2_(dba)_3_ (3.4 mg, 2 mol %) and P(*o*-tolyl)_3_ (8 mg, 9 mol %) were added and purged with nitrogen gas for 25 minutes. The reaction mixture was heated at 90 °C for 30 min. The polymer solution was then added to methanol and the solid formed was collected by filtration. The polymer was re-dissolved in chloroform by heating at 60 °C for 1 h and 10% aqueous solution of sodium diethyldithiocarbamate trihydrate (100 mL) was added and stirred at room temperature overnight. The chloroform soluble portion was separated and washed with distilled water three times. The chloroform solution was reduced to small volume and then added to methanol. The solid was collected and then purified by soxhlet extraction using methanol, hexane, acetone and diethyl ether. Finally, the polymer that goes into chloroform was collected and the volume was reduced and precipitated by adding on methanol. The polymer was collected by filtration, dried in vacuum oven at 40 °C overnight to give a brown solid (167 mg).

### Device fabrication and characterization

All materials, PEDOT:PSS (poly(3,4-ethylenedioxythiophene):poly(4-styrenesulfonate), Clevios P VP A1 4083, H.C. Starck), PC_61_BM ([6,6]-phenyl-C61-butyric acid methyl ester, Solenne BV), anhydrous 1,2,4-trichlorobenzene (TCB) and 1,2-dichlorobenzene (ODCB) were purchased from commercial sources (Sigma-Aldrich) and used without further purification.

Analogously to the description in [21] we report the main steps for the preparation and characterization of the devices. Patterned ITO-coated glasses (*Rs* ~ 10 Ω sq^−1^) were cleaned in sequential sonicating baths (for 15 min) in deionized water, acetone and isopropanol. After the final sonication step, substrates were dried with a stream of Ar gas and then placed in an oxygen plasma chamber for 5 min. Next, a thin layer (~30 nm) of PEDOT:PSS was spun-cast on the ITO surface and subsequently annealed at 150 °C for 15 min. The active layer blend solutions were formulated inside the glove box and stirred overnight at 80 °C. The active layers were prepared from solutions of PTzBDT-1:PC_61_BM and PTzBDT-2:PC_61_BM, dissolved in ODCB or TCB with a total concentration of 36 mg/mL. The resulting solutions were deposited in a glove-box by spin-coating on top of the ITO/PEDOT:PSS surface. Before cathode deposition, always in a glove-box, the substrates were then either thermally annealed or left as-cast. To complete the device fabrication, LiF and Al (0.6 and 100 nm) were deposited sequentially without breaking vacuum (~1 × 10^−6^ Torr) using a thermal evaporator directly connected to the glove box. The current–voltage (*J*–*V*) characteristics of all devices were recorded by a Keithley 236 source-measure unit under AM1.5G simulated solar irradiation, 100 mW/cm^2^ (Abet Technologies Sun 2000 Solar Simulator). The light intensity was determined by a calibrated silicon solar cell fitted with a KG5 color glass filter to bring spectral mismatch to unity. The active area of the solar cell was exactly 6 mm^2^. During testing, each cell was carefully masked, by calibrated mask, to prevent an excess photocurrent generated from the parasitic device regions outside the overlapped electrodes area. All solar cells were tested, without encapsulation, inside the glove box in oxygen and moisture free environment.

### Thin-film characterization

All thin-film characterizations were performed in air. Film optical absorption spectra were recorded on a JASCO V-550 spectrophotometer. The thickness of the various active layers was measured by a profilometer (KLA Tencor, P-6). Atomic force microscopy (AFM) images, recorded directly on tested devices, were taken with a Solver Pro (NT-934 MDT) scanning probe microscope in tapping mode.
